# K_2_O-Metakaolin-Based Geopolymer Foams: Production, Porosity Characterization and Permeability Test

**DOI:** 10.3390/ma15031008

**Published:** 2022-01-27

**Authors:** Elettra Papa, Elena Landi, Francesco Miccio, Valentina Medri

**Affiliations:** National Research Council of Italy, Institute of Science and Technology for Ceramics (CNR-ISTEC), Via Granarolo 64, 48018 Faenza, Italy; elena.landi@istec.cnr.it (E.L.); francesco.miccio@cnr.it (F.M.)

**Keywords:** geopolymer foam, direct foaming, porosity, permeability

## Abstract

In this paper, four near-net shaped foams were produced via direct foaming, starting from a benchmark metakaolin-based geopolymer formulation. Hydrogen peroxide and metallic silicon were used in different amounts as blowing agents to change the porosity from meso- to ultra-macro-porosity. Foams were characterized by bulk densities ranging from 0.34 to 0.66 g cm^−3^, total porosity from 70% to 84%, accessible porosity from 41% to 52% and specific surface area from 47 to 94 m^2^ g^−1^. Gas permeability tests were performed, showing a correlation between the pore features and the processing methods applied. The permeability coefficients k_1_ (Darcian) and k_2_ (non-Darcian), calculated applying Forchheimer’s equation, were higher by a few orders of magnitude for the foams made using H_2_O_2_ than those made with metallic silicon, highlighting the differing flow resistance according to the interconnected porosity. The gas permeability data indicated that the different geopolymer foams, obtained via direct foaming, performed similarly to other porous materials such as granular beds, fibrous filters and gel-cast foams, indicating the possibility of their use in a broad spectrum of applications.

## 1. Introduction

Ceramic foams have been receiving increasing interest due to their exceptional combination of properties that include temperature and corrosion resistance, their low weight, low thermal conductivity, the high permeability and tortuosity of flow paths, high specific surface area, and so forth [1].

At present, macro-porous ceramics have been widely applied in different technological areas, for instance, in thermal and acoustic insulation, pre-cast building materials, sound adsorption and noise reduction, as catalyst carriers and in wastewater treatment, high-temperature exhaust gas filtration and corrosive gas filtration, among others [2,3,4].

In line with net-zero emissions strategies, the development of foams from sustainable raw materials and using low energy production processes is of particular interest. In this context, geopolymer foams have some advantages in comparison with ceramic foams. Indeed, geopolymers are synthetic alkaline alumino-silicate inorganic polymers obtained at a temperature below 100 °C through a chemical reaction between a highly alkaline aqueous solution and a powder containing silicon and aluminum elements [5,6], as well as from waste materials. Furthermore, foaming methods often used for ceramics can also be adopted for geopolymers.

For this reason, geopolymer foams have been the focus of attention in the field of eco-friendly porous materials, also thanks to their low shrinkage after forming, advantageous thermomechanical resistance and chemical similarity to ceramics, together with their zeolite-like properties, and so on [7]. They have been employed as thermal and acoustic insulators, membranes and catalyst supports, for photocatalytic degradation applications and as heavy metal and dye adsorbents, to give just a few examples [8].

It is important to mention that geopolymers have an intrinsic porosity of around 35–40%, with nanopores in the mesoporous range (2–50 nm). Their final amorphous to semi-crystalline 3D network is composed of nanoprecipitates separated by pores [9,10]. Therefore, foaming methods allow the widening of the pore size range up to and over the macro-porous region (pores > 50 nm), even reaching the millimetric scale.

Although porous geopolymers have also been produced using sacrificial template methods [11,12,13,14], additive manufacturing [15] and spherification [16,17,18], direct foaming is the most used method, and several examples are present in the literature exploring different blowing agents and surfactants [19,20,21]. For example, hydrogen peroxide, aluminium and silicon metallic powder have been extensively used as blowing agents for the fabrication of geopolymer foams [22].

Blowing agents are able to generate gas bubbles in the starting geopolymer slurry. Porous materials are obtained when bubbles are incorporated into the suspension that subsequently sets, maintaining the structure generated by the bubbles. In general, the amount of gas trapped in the liquid medium affects the total porosity of the foamed material, whereas the pore size is determined by the stability of the wet foam before setting. During the direct foaming process, it is important to stabilize the air bubbles generated in the liquid slurry and surfactants and particles are often used for this purpose.

As regards the final applications, the correlation of porosity with the permeability of a porous medium plays a vital role in processes involving filtration, fluid mixing, chemical reaction, mass transfer or heat transfer [2,23]. Indeed, permeability is influenced by pore throat size and the interconnectivity of the pore system [1] and it is regarded as a macroscopic measure of the ease with which a fluid driven by a pressure gradient flows through the voids of a porous medium. Thus, permeability reflects the interaction between the fluid and the porous medium [2]. In order to be permeable, the internal porosity of the medium should be well accessible, with a percolation threshold above a critical value, depending on the shape and distribution of pores [24].

In this context, geopolymer foams were produced via direct foaming with different types and percentages of porosity to obtain knowledge about foams’ microstructural parameters and porosity, with a special attention paid to the characterization of their permeability. Because direct foaming significantly affects the viscosity of the reaction slurry, the rheology of the system and, by extension, the whole geopolymerization process, the intrinsic and induced porosities of foamed geopolymers are affected as well [10]. The reason for this is that the reactions driven by blowing agents either consume or release water [20], changing the water content in the starting slurry, namely, the liquid/solid ratio (L/S). In particular, K_2_O-metakaolin-based geopolymer foams were successfully synthesized, adding two blowing agents, i.e., metallic silicon powder and hydrogen peroxide.

The silicon metal powder reacts in the alkaline geopolymer slurry following the redox reaction (1), with the evolution of hydrogen bubbles and the consumption of water (the geopolymer reaction medium) [10].

Si_0_ + 4H_2_O → 2H_2_↑ + Si(OH)_4_ (1)

Therefore, Si affects the induced porosity of geopolymers, as well as the intrinsic porosity. The water-consuming process increases the viscosity of the slurry, modifying the rheology of the system. Furthermore, Si increases the Si/Al ratio, the key factor for the geopolymerization process, giving the system more elastic features [20].

Conversely, hydrogen peroxide, an oxidizing agent, decomposes under ambient conditions following reaction (2), releasing water and oxygen gas, which results in the formation of pores.

2H_2_O_2_ → 2H_2_O + O_2_↑ (2)

Because hydrogen peroxide is usually added as an aqueous solution to the geopolymer slurry and because of the release of water, the viscosity of the starting mixture is modified, and pore formation and the pore size distribution are consequently affected [20].

In addition, a composite foam was produced using alumina as a filler. In general, the use of inert fillers and fine particles, such as α-Alumina (Al_2_O_3_), are often reported as reinforcements to provide higher mechanical strength, reducing the shrinkage of the geopolymer binder [25,26]. Since the highly crystalline nature of α-Al_2_O_3_ can prevent it from participating in the geopolymerization reaction, it can act as an inert filler, affecting the rheology of the slurry and consequently the foam expansion, the porosity and the final mechanical properties [27].

Although many studies have been carried out to design geopolymer foams for specific applications, information on the impact of manufacturing parameters on porosity, pore nucleation and foam properties has often been missing, and this paper aims to address this research gap.

Foams were fully characterized in terms of their macro- and microstructure, pore size distribution, specific surface area and chemical composition to enable the discussion of the effects of blowing agents on the intrinsic and induced porosity and gas permeability findings. Permeability tests with nitrogen were performed and Darcian and non-Darcian permeability constants were calculated to classify the behavior of the geopolymer foams in comparison with other conventional ceramic materials, indicating a possible use for them in a broad spectrum of applications.

## 2. Materials and Methods

### 2.1. Foam Preparation

A planetary centrifugal mixer (THINKY MIXER ARE-500, THINKY CORPORATION, Tokyo, Japan) was used to obtain a benchmark geopolymer slurry (coded G), suitable to produce consolidated geopolymers with theoretical molar ratios SiO_2_:Al_2_O_3_ = 4.0 and K_2_O:Al_2_O_3_ = 0.8. The slurry was prepared via mixing for 3 min at 900 rpm of metakaolin (Argical™ M1200S, Imerys, Paris, France, SSA = 25 m^2^ g^−1^, d_50_ = 1.5 mm) with a potassium di-silicate solution (H_2_O:K_2_O = 13.5, SiO_2_:K_2_O = 2.0 molar ratio). The alkaline solution was previously obtained by dissolving KOH pellets (>85%, Sigma Aldrich, St. Louis, MO, USA) and fumed silica (99.8%, Sigma Aldrich) in deionized water.

The foams were then obtained via the addition of two different foaming agents. In one case, metallic silicon powder (grade AX10, H.C. Starck, d_50_ = 4.5 µm) was blended with G slurry in different percentages (0.03 or 0.05 wt.% over G slurry), using the planetary centrifugal mixer for a further 30 s at 900 rpm. A composite foam was produced by adding a 9 wt.% of α-Al_2_O_3_ (CL3000SG, Alcoa, Pittsburgh, PA, USA, S.S.A. = 1.0 m^2^ g^−1^, d_50_ = 3.6 µm) to the starting G slurry and mixing with the planetary mixer for 3 min at 900 rpm. Then, silicon was added and mixing was performed for 30 s at 900 rpm.

In the second case, hydrogen peroxide (Sigma Aldrich, 35 vol.%) in different percentages (3 or 5 wt.% over G slurry) was added to G slurry and then manually mixed for 30 s to homogenize.

All slurries were cast into cylindrical open plastic moulds, with a diameter of 2.7 cm and a height of 15 cm, to allow for the expansion of the foams. The foams were cured for 24 h in a heater set at 60 °C.

The different foam codes and the blowing agent used, as well as the amount used, are reported in Table 1, together with the liquid/solid (L/S) ratio, calculated including the blowing agents.

### 2.2. Characterization of the Foams

Geopolymerization was evaluated through attenuated total reflection (ATR) measurements, obtained using a Thermo Scientific Nicolet iS5 FTIR Spectrometer equipped with an iD7 ATR accessory and diamond crystal. Each spectrum was accumulated from 32 individual measurements, in the acquisition range of 4000–400 cm^−1^, performed on pulverized foams.

The bulk density of the foams was calculated using the weight-to-volume ratio. The foam’s geometrical volume was calculated using calipers (accuracy ± 0.05 mm). The true density of the material was obtained by analyzing the powdered foams with a helium pycnometer (Multivolume pycnometer 1305, Micrometrics).

The total porosity of the foam was then calculated according to Equation (3):

Total porosity (%) = (1 − (bulk density/true density)) × 100(3)

The foam expansion was calculated according to Equation (4):

Foam expansion (%) = ((h_2_ − h_1_)/h_1_) × 100 (4)

where h_1_ is the initial height of the geopolymer slurry cast in the plastic mould and h_2_ is the final height reached by the consolidated foam.

The microstructural features of the foams were examined using an Environmental Scanning Electron Microscope (E-SEM FEI Quanta 200, FEI, Hillsboro, OR, USA). The samples were previously made conductive by applying a thin gold layer using a turbo-pumped sputter coater (Quorum Q150T ES, Quorum Tech, Laughton, United Kingdom).

The dimensional distributions of the macro-pores of the foams were obtained by analyzing high-resolution images with the open-access ImageJ software [28]. Feret diameters were used for the macro-pore distributions.

Pore size distribution, in the range of 0.0058–100 µm, was analyzed via mercury intrusion porosimetry (MIP) (Thermo Finnigan Pascal 140 and Thermo Finnigan Pascal 240, Thermo Fisher Scientific, Waltham, MA, USA), analyzing millimetre-sized fragments coming from half of the heights of the geopolymer foam cylinders.

The measurement of the specific surface area (SSA) was carried out on the foams using a Thermo Scientific™ Surfer instrument, Thermo Fisher Scientific, Waltham, MA, USA. The specific surface area was calculated using the Brunauer–Emmett–Teller (BET) method, by means of nitrogen adsorption at 77 K.

### 2.3. Permeability Tests

Permeability tests were carried out in a laboratory plant consisting of an electronic mass-flow meter and controller (Brooks mod. SLA-5850, Brooks Instrument, Hatfield, PA, USA) for supplying a tuneable stream of nitrogen, a switching valve, a sample holder (1” stainless steel tube) and an electronic differential manometer (AMECAL ST-8890, AML Instruments, Lincoln, United Kingdom) to measure the pressure drop between the inlet and at the outlet of the sample.

For the test, the foams with a diameter of 2.6 cm and a height around 3.5 cm were sealed in the stainless steel sample holder using a thin layer of silicone glue.

The permeability of nitrogen through the porous samples was expressed considering Forchheimer’s equation for compressible fluids (5) [2]:

(5) $$\frac{P_{i}^{2}-P_{o}^{2}}{2\;P_{o}L}=\frac{\mu }{k_{1}}\;\nu_{s}+\frac{\rho }{k_{2}}\;\nu_{s}^{2}$$

where *P_i_* is the gas pressure at the inlet and *P_o_* the one at the outlet of the sample with thickness *L*, measured as a function of the fluid velocity *ν_s_*, calculated with respect to the open section of the tube.

The collected data were fitted according to the least-squares method to a parabolic model y = *a*x + *b*x^2^, where:

y is $\frac{P_{i}^{2}-P_{o}^{2}}{2\;P_{o}L}$, and x is the fluid velocity *ν_s_* [2].

The permeability parameters were then calculated from the model constants *a* and *b* as k_1_ = µ/*a* and k_2_ = *ρ*/*b*, considering nitrogen viscosity and density at room temperature. These parameters are defined as Darcian (k_1_) and non-Darcian (k_2_) permeabilities, respectively.

## 3. Results and Discussion

### 3.1. ATR-FTIR Measurements: Geopolymerization of Foams

The foam formation process is based on geopolymerization and foaming at the same time. Therefore, these reactions must be balanced to obtain a consolidated and reacted geopolymer foam.

**Figure 1:**
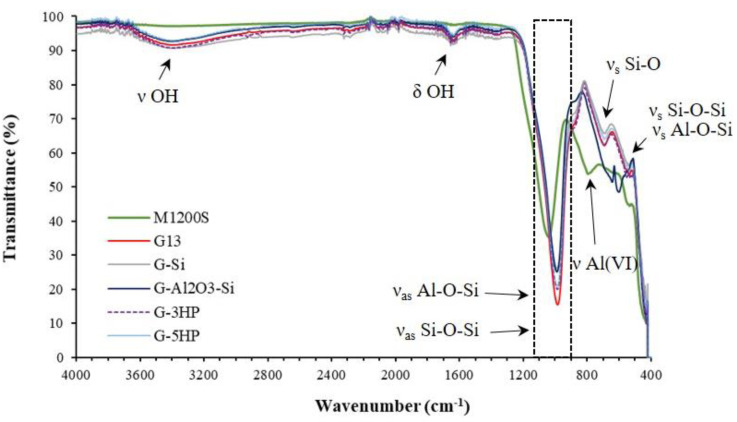
ATR-FTIR spectra of the foams G-Si, G-Al_2_O_3_-Si, G-3HP and G-5HP, compared with the spectra of the geopolymer matrix G13 [10], used as reference, and with the metakaolin M1200S used as a precursor.

In Figure 1, the ATR-FTIR spectra of the foams are reported and compared with a benchmark geopolymer matrix (G13), which was previously characterized and used as a reference for completely reacted geopolymer [10], and with metakaolin M1200S used as a precursor. All the foams’ spectra were superimposable, except for G-Al_2_O_3_-Si, which showed two peaks at 642 and 599 cm^−1^, due to the presence of α-Al_2_O_3_ [29]. In particular, the bands around 3400 cm^−1^ and 1654 cm^−1^ are attributed to the stretching of the O-H water bound (*ν* OH) and to the water bending (*δ* OH), respectively [30,31]. Indeed, water molecules are surface-adsorbed or remain entrapped in the large cavities of the geopolymer framework [32].

The main band, concerning the asymmetric stretching vibrations of Si–O–Si and Al–O–Si, appeared as a broad band between 1100 and 900 cm^−1^ (*ν_as_* Si-O-Si, Al-O-Si). This band, centred at 1042 cm^−1^ for metakaolin, was shifted to a lower wavenumber (986–988 cm^−1^) in the spectra and for the reference geopolymer matrix G13, indicating the formation of the amorphous aluminosilicate gel phase because of the geopolymerization [32].

Therefore, the foaming methods did not negatively affect the geopolymerization. In fact, metakaolin is dissolved by the alkaline solution during the geopolymerization and the species derived from the hydrolysis polymerize, forming a solid network [30]. The shift correlated to this band confirms the dissolution of the starting metakaolin and the polycondensation, which cause the rearrangement of the Si-O-Si and Si-O-Al bonds in the foams [33].

The band at ≈792 cm^−1^, related to stretching vibration of 6-fold coordinated Al(VI) in metakaolin (*ν* Al(VI)), disappeared after polymerization in the geopolymer spectra, replaced by the presence of a band at ≈690 cm^−1^ ascribed to Si–O symmetrically stretching (*ν_s_* Si-O). This showed that 6-coordinated Al(VI) changed into a 4-coordinated one, and participates in the framework structure [34]. The band at ≈ 540 cm^−1^ was ascribed to the symmetric stretching of Si–O–Si and Al–O–Si and it is present only in the geopolymer spectra (*ν_s_* Si-O-Si, Al-O-Si) [32].

### 3.2. Macro-Microstructure of the Foams and Porosity

**Figure 2:**
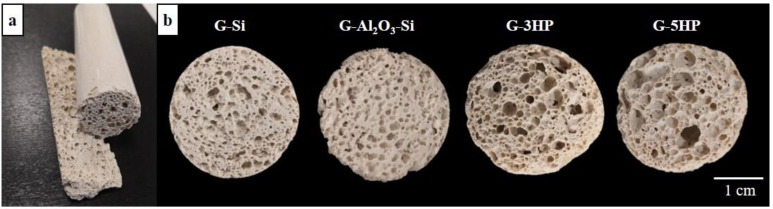
(**a**) G-5HP tube and its longitudinal section. (**b**) Cross sections of the foams.

Slurries and foaming were optimized following a trial-and-error approach to obtain near-net-shaped porous tubes with a size of 2.6 cm diameter and a height 3.5 cm, which were already suitable for the permeability test, as shown for G-5HP sample in Figure 2a.

Cross sections of the foams are shown in Figure 2b, whereas expansion, bulk and real density, as well as the calculated total porosity percentage are reported in Table 2. In general, the mentioned properties and the foam morphology mostly depended on the blowing agent and the water content (L/S ratio) of the slurry (Table 1), as well as the shape of the mold [35].

The starting L/S was set to favor the geopolymerization reaction and the foaming at the same time. As in this work, the water content had to be kept at the lowest quantity to have enough alkalinity and workability for the formation of intrinsic and induced pores, it follows that the foaming process generates bubbles that can be trapped within the matrix, avoiding as much as possible connecting with each other and becoming coarser. However, hydrogen peroxide is water-producing, whereas metallic silicon is water-consuming. It follows that, compared to a rather similar starting L/S ratio (Table 1), foams obtained using Si exhibited a lower expansion with a consequent higher bulk density around 0.7 g cm^−3^ and a lower total porosity% (Table 2), whereas foams obtained using hydrogen peroxide were more lightweight, with a density around 0.3 g cm^−3^.

Observing the cross sections in Figure 2b, it is evident that the blowing agent affected the shape and dimension of the millimetric macro-pores. The increase in H_2_O_2_ from 3 to 5 wt.% seemed not to drastically affect the total porosity and the bulk density of the foams. However, pores tended to coalesce, forming some isolated bigger ultra-macro-pores, as visible in Figure 2b, for G-5HP. Conversely, a correct balance between the silicon amount and the L/S ratio led to the formation of a more homogeneous pore morphology and dimensions for the foams G-Si and G-Al_2_O_3_-Si, as shown in Figure 2b. The use of such a low content of blowing agent allowed the slowing down of the Si redox reaction (1), which progressed synchronously with the geopolymerization.

**Figure 3:**
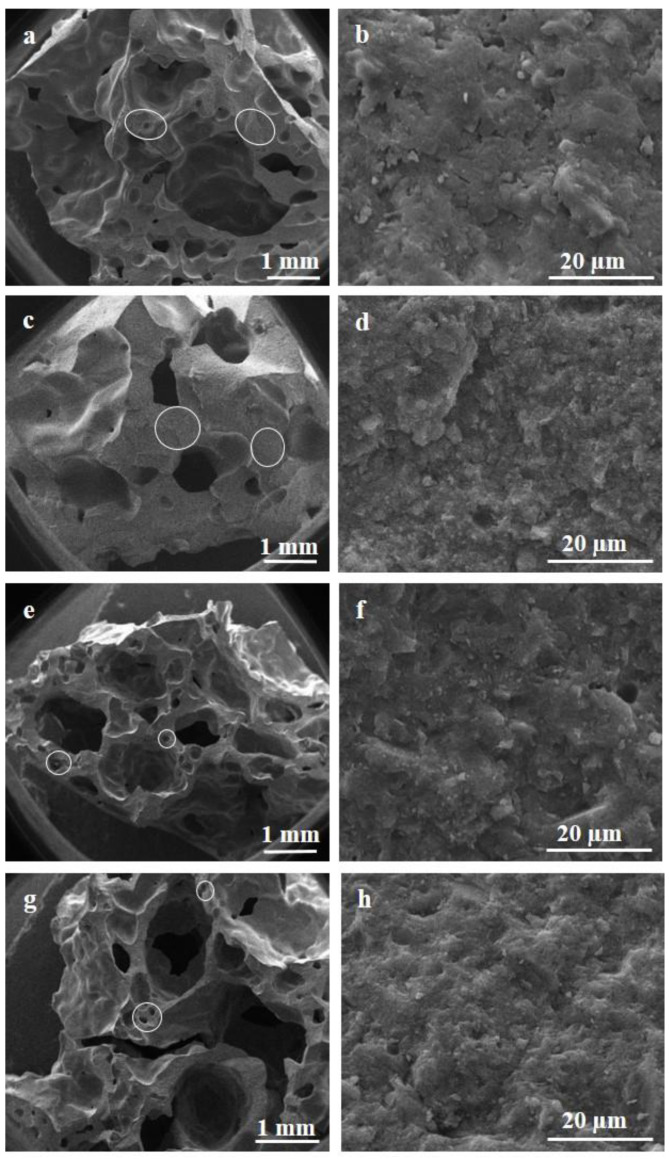
SEM micrographs of foams G-Si (**a**,**b**), G-Al_2_O_3_-Si (**c**,**d**), G-3HP (**e**,**f**) and G-5HP (**g**,**h**). Interpore partitions are indicated by circles.

SEM micrographs of the foams are illustrated in Figure 3. At low magnification (Figure 3a,c,e,g) the macrostructure of the foams is evident, generally consisting of ultra-macro-pores of millimeters in size. At high magnification, all the foams show a rather compact pore-wall microstructure (Figure 3b,d,f,h) and the typical geopolymer precipitates are visible, confirming that a geopolymerization reaction occurred.

Because of the higher expansion with H_2_O_2_, the foams G-3HP and G-5HP (Figure 3e,g, respectively) showed bigger pores and reduced interpore partitions. These interpore partitions however were compact and formed by smaller macro-pores. Conversely, G-Si and G-Al_2_O_3_-Si foams showed rounded and more uniform pores and wider interpore partitions (Figure 3a,c, respectively). The addition of Al_2_O_3_ as a filler further increased the viscosity of the slurry, resulting in a uniform final foam with small and rounded pores (Figure 2b and Figure 3c). G-Al_2_O_3_-Si foam was the least expanded (Table 2), with denser interpore partitions (Figure 3c).

**Figure 4:**
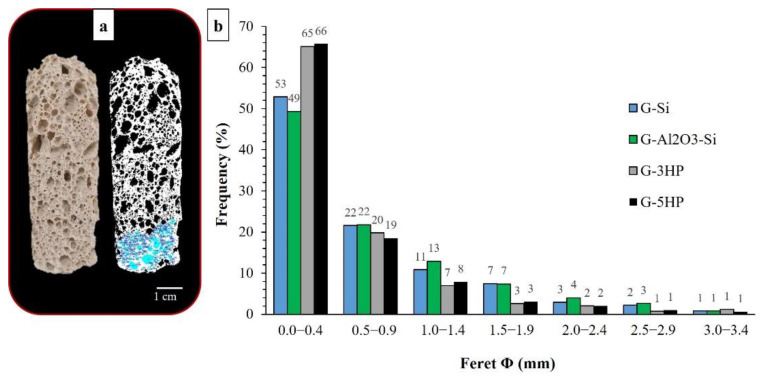
(**a**) Longitudinal cross section of G-5HP and its elaboration for analysis through ImageJ software. (**b**) Feret pore diameter distributions of the foams.

Furthermore, the geometry of the mold affects the expansion and the formation of the pores, in particular the ratio between the opening surface, the height of the mould and the initial volume of the mixture. In the case of H_2_O_2_, the decomposition reaction (1) was instantaneous, with a faster gas evolution. As mentioned before, the reaction releases water and the pore structure in the monolith varied according to the change in the viscosity of the reaction system. The longitudinal section of these foams, as reported for G-5HP in Figure 4a, showed that larger pores were present on the upper part of the foam, due to the coalescing phenomena of gas bubbles moving towards the escape surface. Conversely, smaller pores were present in the bottom, where sedimentation phenomena occurred, hindering the coalescence of gas bubbles and their escape, so the foam was more compact.

The ultra-macro-porosity (>100 µm) of the foams was investigated via image analysis, whereas the pore size distribution in the smaller pore range, mostly accounting for the intrinsic porosity of the geopolymer matrix, was obtained via MIP. Since the foams showed the formation of irregular ultra-macro-pores, as evidenced in the cross sections in Figure 2b and in the SEM micrographs reported in Figure 3, the maximum Feret diameter was considered for the corresponding pore-size distributions reported in Figure 4b. Indeed, the Feret diameter, calculated using the program ImageJ, is defined as the longest distance between any two points along the selection boundary [28]. The distributions were obtained by analyzing the longitudinal sections of the foams, after processing the images as shown in Figure 4a for sample G-5HP, used as example. Most of the pores fell within 3 mm; however, some sporadic larger pores, up to 7 mm, were found in particular for foams G-3HP and G-5HP. In fact, as mentioned before, H_2_O_2_ decomposition is instantaneous and vigorous and generates water, allowing the easier formation of bubbles with the possibility of coalescence. The blowing agent clearly affected the fresh paste and, consequently, the morphology of the hardened foams; despite the presence of some bigger macro-pores, these foams showed the presence of a greater number of macro-pores, of which the partitions were formed of smaller pores, as evidenced by the SEM micrographs shown in Figure 3e,g. In general, distributions showed that the pores with diameters within 0–0.5 mm accounted for around 65%, followed by 20% for the pores between 0.5–1 mm, 8% for pores between 1–1.5 mm and the remaining 7% for pores > 1.5 mm (Figure 4b).

G-Si and G-Al_2_O_3_-Si foams displayed a lower pore count; the Feret distributions accounted for ≈ 50% of the pores between 0–0.5 mm, 22% between 0.5–1 mm, ≈12% between 1–1.5 mm and the remaining 16% for pores > 1.5 mm. These foams presented a higher percentage of pores larger than 1.5 mm, but pores were mostly concentrated in the range of 1.5–3 mm, unlike G-3HP and G-5HP, which showed the presence of pores above 4.5 mm because of coalescence phenomena.

**Figure 5:**
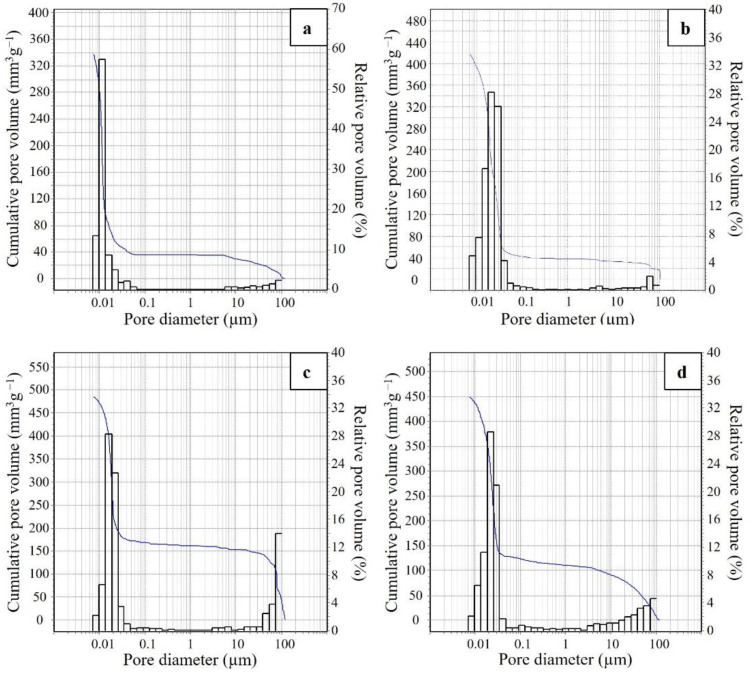
Pore size distributions by MIP of (**a**) G-Si, (**b**) G-Al_2_O_3_-Si, (**c**) G-3HP and (**d**) G-5HP.

The porosity of the cell struts in the range of 0.0058–100 µm was investigated via MIP analysis and pore size distributions are reported in Figure 5, whereas the accessible total porosity %, total pore volume and modal pore diameter are reported in Table 2. The distributions mainly accounted for the small accessible pores derived from alkali-activation and polycondensation reactions that occurred in the foams, and for the smaller macro-pores resulting from the foaming reactions.

In general, foams had similar accessible total porosity percentages by Hg intrusion, comprising between 41% and 52% (Table 2), with a modal pore diameter that slightly increased from 0.01 µm for G-Si, to 0.02 µm for G-Al_2_O_3_-Si and G-3HP, up to 0.03 µm for G-5HP (Figure 5). Observing the distributions, most of the pores were located between 0.007 and 0.1 µm, with 88% for G-Si, 90% for G-Al_2_O_3_-Si, 67% for G-3HP and 73% for G-5HP. However, foams G-3HP and G-5HP (Figure 5c,d, respectively) showed a consistent presence of pores also at greater pore size intervals, especially in the range of 10–100 µm, attributable to the use of the foaming method with H_2_O_2_.

Regarding the specific surface area calculated using the BET method (Table 2), G-Si showed the highest value of 94 m^2^ g^−1^, because the low amount of Si did not alter the slurry formulation, leading to a result similar to a fully reacted geopolymer with the same stoichiometry [10]. The specific surface area of G-Al_2_O_3_-Si decreased to 65 m^2^ g^−1^ because of the presence of alumina as a filler, with a low specific surface area of 1 m^2^ g^−1^ and a mean diameter of 3.6 µm.

Regarding the foams obtained with H_2_O_2_, there was not a clear correlation between the specific surface area values. Indeed, the low value for G-3HP (47 m^2^ g^−1^) could be explained considering that the slurry was diluted by the addition of H_2_O_2_ solution and by the water remaining after the decomposition reaction. When the L/S ratio is high, the geopolymerization reaction is affected accordingly, as a high water content slows down the polymerization with the consequent formation of bigger precipitates that cause a lowering of the specific surface area [10,36]. However, the higher value registered for G-5HP (81 m^2^ g^−1^) was not part of this trend, with the L/S being higher than that in G-3HP. It can be supposed that the increase in the specific surface area of G-5HP was due to the highly exothermic decomposition reaction of hydrogen peroxide, with a standard enthalpy of −95 kJ mol^−1^ [37]. The increase in the H_2_O_2_ content probably determined a greater development of heat that, combined with the subsequent treatment at 60 °C, could have favored the geopolymerization. Indeed, the literature reports that an increase in temperature favors the formation of highly reactive geopolymers [38,39].

### 3.3. Permeability Test

The permeability measurements, according to Forchheimer’s Equation (5), described the resistance of the gas flow (N_2_) through the porous foams, considering the influence of both viscous and inertial effects on the pressure drop [1].

**Figure 6:**
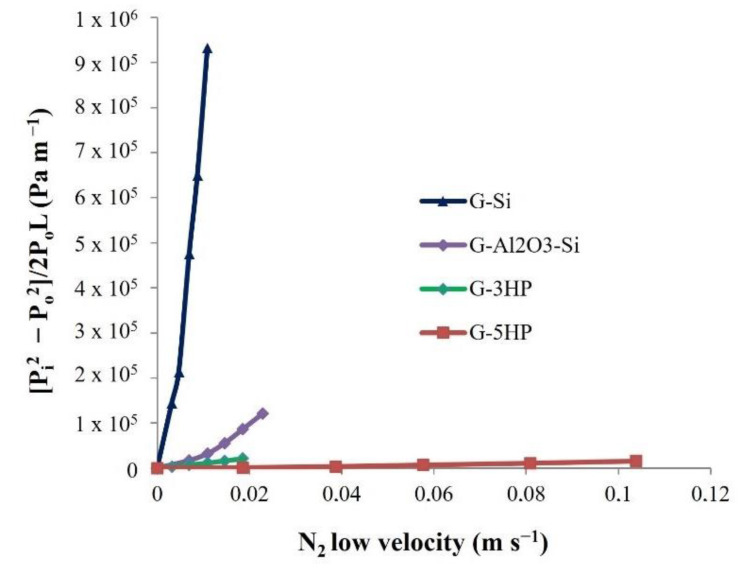
Pressure drop curves obtained at room temperature for the geopolymer foams.

The experimental pressure drop curves are shown in Figure 6, whereas the calculated k_1_ (Darcian) and k_2_ (non-Darcian) permeabilities are reported in Table 3. These parameters are considered to be dependent only on pore characteristics (size, pore count, interconnectivity, strut thickness) [1,2]. Indeed, permeability is expected to change as a function of several parameters of the foams that reflect different aspects of the fluid-solid interaction [2]. In the conducted test, nitrogen flow loses energy due to friction with the porous wall; therefore, the higher the contact area, the greater the resistance to gas flow [40]. For porous materials, the increase in the contact area could be associated with a reduction in the pore size [41]. Furthermore, changes in the processing parameters that favor the development of interconnected porosity and the increase in pore size, or which decrease the pore tortuosity and roughness, increase the permeability with consequently higher values of k_1_ and k_2_ [40].

Concerning the permeation behaviour of the foams, it is evident that the k_1_ and k_2_ coefficients were higher by several orders of magnitude for the foams obtained using H_2_O_2_ (Table 3). These foams showed a higher total and open porosity percentage (Table 2) and the presence of larger pores with thinner pore struts, which were highly porous in turn, as evidenced by SEM micrographs (Figure 3e,g). The interconnectivity of these foams was increased, reducing the N_2_ flow friction, therefore leading to higher values for k_1_ and k_2_.

The higher amount of H_2_O_2_ in G-5HP, compared to G-3HP, led to the formation of isolated larger pores, as shown in Figure 2b, which probably promoted the gas permeability, increasing the constants by one order of magnitude (Table 3).

Foams G-Si and G-Al_2_O_3_-Si showed lower values of k_1_ and k_2_. These foams expanded less with lower total porosity and, more importantly, with thicker pore struts (Figure 3a,c), which increased the friction of the gas. Moreover, these foams had smaller and rounded pores, which could increase the contact area and the tortuosity.

The higher k_1_ and k_2_ values of G-Al_2_O_3_-Si, compared with G-Si, may be due to the higher open porosity (Table 2), which increased the gas accessibility [40].

Geopolymer foams can be compared with other porous materials using the permeability map developed by Innocentini et al. in [2], which correlates the values of viscous (k_1_) and inertial (k_2_) constants and classifies porous materials according to their permeability and application.

**Figure 7:**
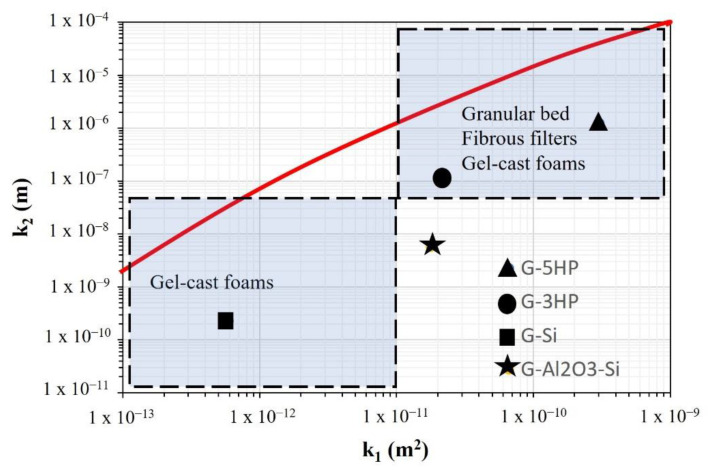
Geopolymer foams’ permeability values, k_1_ and k_2_, located on the adapted permeability map from [2]. The red line represents the best fit (k_2_ = exp(−1.71588 k_1_^−0.08093^)) considering the whole data set collected in literature [2].

In Figure 7 the adapted version of this map is shown, including the k_1_ and k_2_ values calculated in this paper and with evidence of the macro-areas in which our geopolymer foams fell.

Foams obtained using H_2_O_2_ (G-3HP and G-5HP) were in the range of granular beds, fibrous filters and gel-cast foams. G-Si fell in the range of gel-cast foams, whereas G-Al_2_O_3_-Si did not find a well-defined location, with a behaviour halfway between those of the foams already mentioned. Indeed, G-Al_2_O_3_-Si has a quite high L/S ratio, which allows an easier expansion, and coalescence phenomena are avoided during foaming because of the presence of micrometric fillers that stabilize the slurry, leading to the formation of uniform and rounded ultra-macro-pores separated by thicker but macro-porous struts.

## 4. Conclusions

In this paper, a benchmark metakaolin-based geopolymer formulation with molar ratios SiO_2_/Al_2_O_3_ = 4.0 and K_2_O/Al_2_O_3_ =0.8 was used to produce four near-net-shaped porous tubes via direct foaming. Two blowing agents were used, i.e., hydrogen peroxide and metallic silicon, in different amounts in order to change their pores from meso- to ultra-macro-porosities. A correlation between permeability and pore features (porosity, pore size, interconnectivity and strut thickness) was found, and this was significantly dependent on the processing method as this affects the structure of the final foams.

Permeability measures were performed and correlated to the foaming processes and final porosities as follows:

- High L/S ratios in the geopolymer slurry increase the permeability of the foams by increasing the total porosity. In general, the foams from hydrogen peroxides, which is added as an aqueous solution and which releases water, are more porous than foams from metallic silicon, which is water consuming.
- The porosity of the struts influences the permeability due to changes in the frictional area. The addition of inert fillers, such as micrometric alumina, stabilizes the foam with the production of uniform pores and struts that favor the interconnectivity and permeability, although the foam has a higher density and lower expansion.
- Concerning the evaluation of permeability, this showed that the produced geopolymer foams are in the range of granular beds, fibrous filters and gel-cast foams (on the permeability map), with these differences caused by changing the blowing agent in the processing method.

These results highlight the fact that, starting from the same geopolymer slurry, small changes in the direct foaming process can greatly affect the porosity and all related structural features, consequently affecting the permeability of the foams. Geopolymer foams are an interesting area of investigation, and a deep understanding of the various foaming processes is fundamental in order to have a controllable production process, allowing for tailored permeability. Therefore, these results, as well as the advantage of using a sustainable and cheap production method, are encouraging in regard to optimizing the processes used to obtain geopolymer foams, covering the entire permeability map and expanding the application range as much as possible. The practical application of studied foams can be found in high-temperature filtration gases (e.g., flue gases and syngas), owing to the refractory nature of geopolymers.

## Figures and Tables

**Table 1:** Code, blowing agent used and amount (wt.%) and liquid/solid ratio of the produced geopolymer foams.

Foam Code	Blowing Agent (wt.%)	Liquid/Solid Ratio
G-Si	Silicon—0.05	0.42
G-Al_2_O_3_-Si	Silicon—0.03	0.51
G-3HP	Hydrogen peroxide—3	0.46
G-5HP	Hydrogen peroxide—5	0.49

**Table 2:** True and bulk density, total porosity % and expansion %, calculated applying (3) and (4), respectively. Porosity %, total pore volume and modal pore diameter calculated via MIP analysis (*) and specific surface area of the foams.

Foam Code	True D (g cm^−3^)	Bulk D (g cm^−3^)	Total Porosity (%)	Expansion (%)	Porosity by Hg * (%)	Total Pore Volume * (mm^3^ g^−1^)	Modal Pore Ø * (µm)	S.S.A. (m^2^ g^−1^)
G-Si	2.22	0.66	70	79	41	338	0.01	94
G-Al_2_O_3_-Si	2.35	0.69	71	57	49	418	0.02	65
G-3HP	2.13	0.35	84	153	52	484	0.02	47
G-5HP	2.16	0.34	84	164	52	449	0.03	81

**Table 3:** Darcian (k_1_) and non-Darcian (k_2_) permeabilities. Calculated applying Forchheimer’s equation.

Foam Code	k1 (m^2^)	k_2_ (m)
G-Si	5.94 × 10^−13^	2.29 × 10^−10^
G-Al_2_O_3_-Si	1.78 × 10^−11^	5.73 × 10^−9^
G-3HP	2.04 × 10^−11^	1.15 × 10^−7^
G-5HP	3.03 × 10^−10^	1.24 × 10^−6^
